# Supplementary material for: Optimising Interventions for Catheter-Associated Urinary Tract Infections (CAUTI) in Primary, Secondary and Care Home Settings
Source: Antibiotics (Basel). 2020 Jul 17;9(7):419. doi: 10.3390/antibiotics9070419 (PMC7399982; doi:10.3390/antibiotics9070419)
Supplement: Supplementary file 1 [file antibiotics-09-00419-s001.pdf]

Supplementary documents

1. Supplementary Document 1: Prisma Chart- Rapid review
2. Supplementary Document 2: Characteristics of included research studies
3. Supplementary Document 3: The frequency of theoretically congruent BCTs in national and research interventions
4. Supplementary Document 4: List of intervention components included in the survey
5. Supplementary Document 5: Relevance and APEASE scores for all intervention components in primary/community care
6. Supplementary Document 6: Relevance and APEASE scores for all intervention components in secondary care
7. Supplementary Document 7: Relevance and APEASE scores for all intervention components in care homes
8. Supplementary Document 8: Search terms- Rapid review
9. Supplementary Document 9: List of most frequently identified barriers[5]

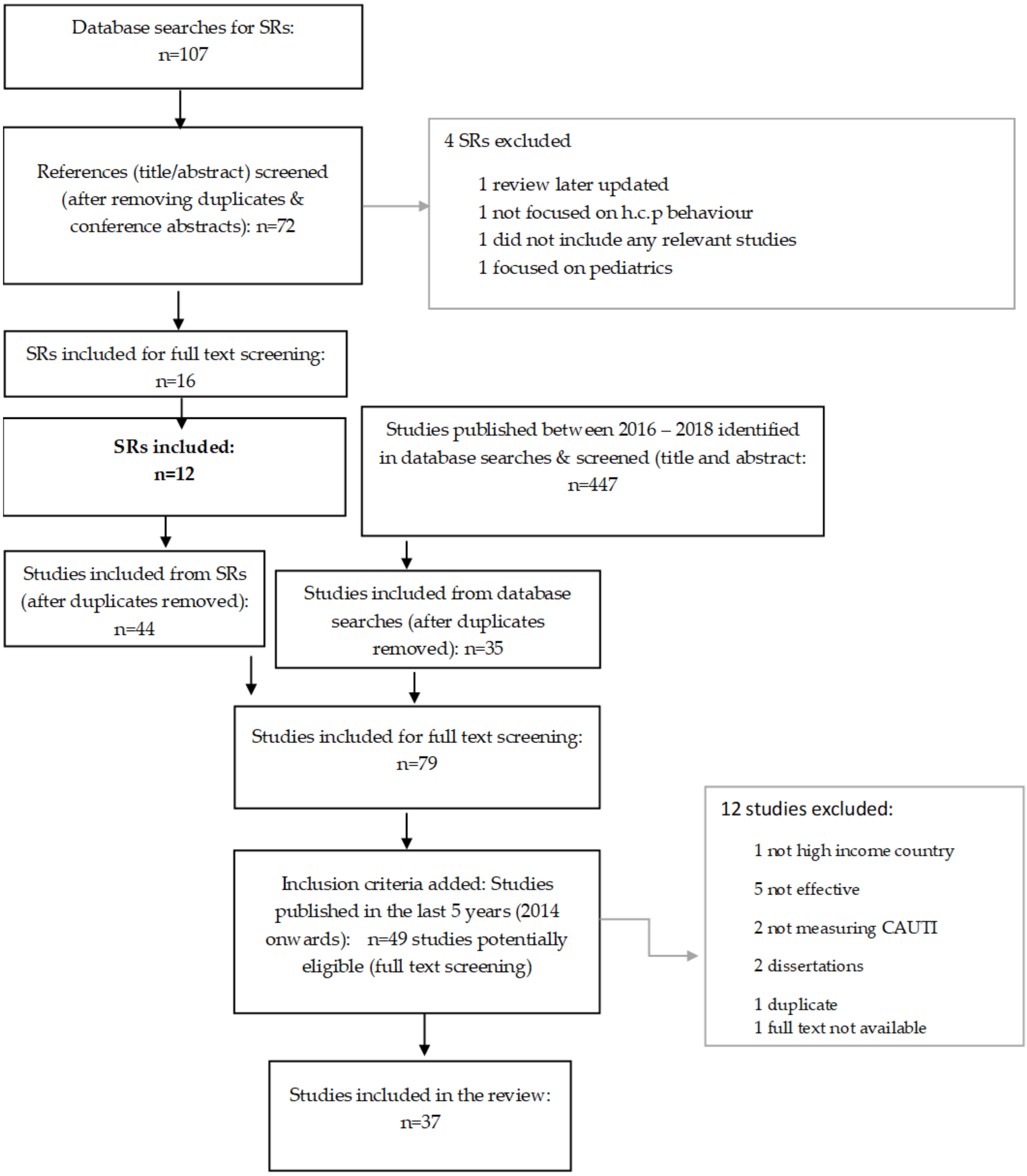

Supplementary Document 1: Prisma Chart- Rapid review.

Supplementary Document 2: Characteristics of included studies**.**

Reference, Country, target group and setting	Study design	Intervention	Outcome	Effectiveness
Bell, M. et al (2016)<br>US; ICU unit in a hospital; Nurses and physicians	Pre-post test	1) Introduction of a CAUTI bundle listing indications for catheters, stressing sterile insertion and appropriate catheter care; introduction of StatLock devices to secure catheters<br>2) Obtaining wicking pads as an alternative to a urinary catheter<br>3) A multidisciplinary continuing medical education conference addressing CAUTI prevention for staff; A sentinel event patient case presented along with information regarding CAUTI, indications for urinary catheters, and strategies to reduce catheter use.<br>4) Medical records modified to create a mandatory drop-down selection box listing the appropriate indications for urinary catheter placement, requiring the provider to select one of the indications from the drop-down menu before they could continue with the urinary catheter order<br>5) Daily tracking of patients with catheters added to the nursing staff daily management system. Numbers posted on a tracking board in each ward and updated daily.<br>6) Implementation of a service tracking patients having urinary catheters and the indications for use.<br>7) Creation of urine retention protocol based on best-practice guidelines approved by the CAUTI systemwide oversight team; protocol added to the hospital electronic health records as a nursing protocol<br>8) Prompt added to the electronic health records reminding physicians each day about patients having catheters and asking whether the catheter could be removed. If the physician clicked no, the system asking for the indication for a continued use.<br>9)The resident quality champion sitting on the systemwide Quality Resource Management Committee, coordinating multidisciplinary continuing medical education conferences, and coordinating patient safety case presentations at monthly resident-faculty meetings.	Incidence of CAUTI	Decrease from pre-intervention period to post intervention period by 25-30% (year 2); no improvement in year 1
Carr et al. (2017);<br>US; a 27-bed Progressive Care Unit at the Mayo Clinic; All staff	Pre-post test	1) Implementation of a CAUTI bundle ensuring standardised approach in catheter use and maintenance involving: a) medical record reviews conducted every shift to ensure an IUC order was written, and indication within CDC (2009) guidelines; b) daily assessment to determine medical necessity of IUCs; c) collaboration with providers to discontinue IUC if no longer appropriate; d) Catheter care every 8 hours and as needed; e) performing hand hygiene, monitoring for kinks in	Incidence of CAUTI	Decrease from pre-intervention incidence of CAUTI of 3 to post-intervention of 0 (sustained for 22 months)

		catheter and tubing, securing IUC appropriately with a leg strap, maintaining integrity of seal between catheter and drainage bag tubing, and ensuring catheter bag is always at least 6 inches above the floor/below the patient's bladder; f) emptying catheter bag every 8 hours or when bag is two-thirds Full g) Accurate documentation in the electronic health record<br>2) In-service sessions concerning use of a unit-wide CAUTI bundle and adherence audit forms developed by the workgroup.<br>3) An RN or PCT initiating the CAUTI Prevention Form for every patient with a newly inserted IUC, or admitted to PCU with existing IUC; The form kept in the patient's room, visible and accessible to staff.<br>4) Use of Cather Audit Form on a weekly basis to review staff documentation of related patient care in order to determine areas of improvement and further education.<br>5) Positive results disseminated widely to increase staff motivation and engagement.<br>6) Team huddles, flyers, and open communication reinforcing the teamwork		
Cartwright, A. (2018); UK; Hospital; All staff	Pre-post test	1) Standardisation of catheters across hospital<br>2) Onsite training by catheter manufacturer to enable correct use of the tray in an aseptic manner<br>3) Continence team visiting all areas of hospital to ensure staff aware of product change and to talk through the contents of catheter pack and answer staff questions.	Incidence of CAUTI	Decrease from pre-intervention in CAUTI incidence of 102 to post-intervention of 54 (year 1), 21 (year 2) and 13 (year 3)
Cho, H. et al. (2017); US; 5 medicine units in a 1,171-bed tertiary Care hospital; hospitalists, other attending physicians, nurse practitioners, and medicine house staff	Pre-post test	1) Development of an electronic health record (EHR) tool to identify catheter use based on nursing documentation.<br>2) The tool used by hospitalist medical director on the medicine units to query providers at daily interdisciplinary rounds whether patient needs a catheter; rounds attended by hospitalists, other attending physicians, nurse practitioners, and medicine house staff<br>3) Education on catheter and CAUTIs added to the monthly orientation for clinicians rotating on the units.<br>4) Development of a mnemonic, NO TUBE, to help providers remember the acceptable indications for catheter use	CAUTI rate per 1000 catheter-days	Decrease from pre-intervention phase in CAUTI rate from 2.85 per 1000 catheter-days to post-intervention of 0.32 per 1000 UC days ($p < .001$)
Davies et al. (2018); US; trauma centre; All staff	Pre-post test	1) Nurse rounds 4 times a week in order to reinforce nurse indications for proper use, maintenance techniques and early discontinuation of catheters; each patient assessed for appropriateness and potential for removal of catheter.	CAUTI rate	Effective in ICU trauma patients only: Decrease from pre-intervention CAUTI rate of 4.09 to post-intervention period of 1.08 (Incidence rate ratio, 0.26; 95% CI, 0.01-1.68);

		2) Creation of a diagnosis algorithm to provide education and supplement bedside rounds<br>3) Prospective surveillance of all new culture analysis by clinical pharmacists and a clinical nurse specialist in order to identify any discordance with guidelines; if that was the case the provider was contacted and provided with education to determine if the urine culture could be cancelled.		
Dawson et al. (2017);<br>UK; district general hospital and major trauma centre; All staff	Pre-post test	1)Implementation of catheter care pathway aimed to consolidate documentation surrounding catheter care, including details of insertion and reviews, and enabling assessment of adherence to the guidelines<br>2) Adherence to pathway monitored in monthly audits by the continence nurse specialist, as part of ongoing quality improvement<br>3) The acronym HOUDINI developed to enable staff to recognise indications for continued urinary catheter use<br>4) HOUDINI checklists added to the front of pathway documentation, to aid staff in their decision-making process at the point of insertion and/or reviews; supported by a series of power training sessions, delivering education and training around the HOUDINI concept directly to staff within the healthcare setting.<br>5) HOUDINI further supported by its inclusion in annual infection prevention link staff study days allowing staff to explore the concept in detail, ask questions and return to their specific units to disseminate the practice.<br>6) All staff invited to enter a competition to design a 'catheter magnet' in order to raise awareness of catheter care; the winning design translated into a magnet and used on patient discharge boards to remind staff to review patient catheters with a view to TWOC (trial without catheter)<br>7) Introduction of bladder Scanners to help identify urinary retention and identify cases where catheter use may not be required; scanners introduced to staff through the use of scheduled demonstrations and training sessions, facilitated by the manufacturers to ensure that the catheter care group and Trust staff aware of the optimal process for using bladder scanners.	Incidence of CAUTI; CAUTI rate	Decrease from early intervention phase in CAUTI incidence of 3.5% to late intervention phase of 2.4%;<br>Average CAUTI rate below the national average (0.38% versus 0.73%).
Dy, S. et al. (2016) ;<br>US; 3 hospitals; Nurses	Pre-post test	1) Creation of evidence-based practice report<br>2) Development of a clinical decision support tool prompting staff to remove catheter<br>3) Education of the staff using online modules, unit-based educational services, Clinical Nurse Specialist day and night rounds<br>4) Appointment of a unit-based clinical nurse "champion" resource<br>5) Standardisation of protocol and policies across hospitals	CAUTI rate per 1000 catheter-days; CAUTI rate per 1000 patient days	Only effective in hospital 1: Decrease from pre-intervention CAUTI rate per 1000 catheter days of 2.25 to post intervention rate of 1.63 (p=0.05);<br>Decrease from pre-intervention CAUTI rate per 1000 patient days of 0.42 to post intervention rate of 0.28 (p=0.02);

		6)Nurse-driven removal protocol (NDPR)set as default; providers having to unselect NDPR		
Kachare et al. (2014) ;<br>US;<br>30-bed surgical oncology unit in a tertiary care centre; All staff	Pre-post test	1) Hospital-wide guidelines published describing the indications for Foley catheters; medical staff encouraged to adhere to them<br>2) Implementation of daily electronic “hard-stop” queries to the primary services through the electronic medical record;<br>3) Direct contact to the primary team as to medical necessity for continued Foley use<br>3) Provision of education and coaching to physicians on recommended best practice and early removal of Foley catheters<br>4) Implementation of Foley removal diagram (guiding management of patients after Foley removal) and guidelines for Foley reinsertions<br>5) Tracking and monitoring of all processes<br>6) Provision of closed drainage system	Median CAUTI rate	Reduction from pre-intervention median CAUTI rate of 4.6 to post-intervention of 0 (p=0.03)
Halperin et al. (20160;<br>US;<br>Neuro ICU; All staff	Pre-post test	1) Re-educating neuro ICU personnel in criteria for appropriateness of catheter placement and removal<br>2) Daily rounds questioning the continued need for catheter<br>3) Introducing a new kit simplifying and standardising sterile insertion; and ensuring that other departments directly related to the ICU followed the same procedure as the ICU. Personnel trained at those departments.<br>4) All ICU staff retrained in Foley catheter insertion by watching a video and performing an observed simulation.<br>5) All RN and patient care technicians retrained in soap and water perineum care, which was then done every 12h shift<br>6) Real time feedback including notification and dissemination of positive changes and maintaining a calendar indicating a number of days since the last CAUTI<br>7) Placing a mobile CT scan in the neuro ICU to reduce transports for brain imagining	Incidence rate of CAUTI per month; CAUTI rate per 1000 catheter-days	Overall decrease from pre-intervention number of CAUTI/month of 2 to post-intervention rate of 0.8;<br>Decrease from pre-intervention CAUTI rate per 1000 catheter days of 10.9 to post-intervention rate of 6.2 (p=0.04)
Johnson, P. et al (2016)<br>US; ICUs; nurses and physicians	Pre-post test	1) Implementation of a nurse-driven catheter removal protocol not requiring additional physician’s ordering the majority of cases. If patient not fitting the protocol, reason for it documented.<br>2) Daily assessment for the need of a catheter during multidisciplinary ICU rounds<br>3) Education of all nurses in the ICUs, chief residents and surgery/medicine physician teams on current CAUTI rates, information about new protocol and evidence guiding its development and review of routine catheter care and maintenance.<br>4) A laminated copy of the order set posted in each unit	Incidence of CAUTI; CAUTI rate	Overall reduction by 36%; Decrease from pre-intervention CAUTI rate of 0.60% to post-intervention rate of 0.43%

		5) Changes in policies and procedures including utilization of smaller bore urinary catheters, and addition of silver-based cleansing products for urinary catheter care		
Maxwell, M. (2018);<br>US; ICU/IMCU in a community hospital; nurses, physicians and nursing assistants	Pre-post test	1) Urine specimen collection only obtained from newly inserted catheters to remove the potential confounding factor of catheter biofilm<br>2) Implementation of a 4-h perineal care and cleaning of catheters every 12hs<br>3) Purchase of female urinals and male urinary devices<br>4) Two nurses required to be present during insertion and then document it<br>5) Approved criteria for catheter insertion added to the insertion order in the electronic records<br>6) Development of evidence-based practice protocols for bladder scanning and catheter removal;<br>6) Use of straight catheterisation encouraged<br>7) Purchase of additional bladder scanners<br>8) Education to the ICU teams<br>9) Catheter assessed daily during interdisciplinary rounds; goals set for anticipated date of catheter removal<br>10) Encouragement provided for the use of other non-invasive catheter alternatives.<br>11) Education provided to all staff about insertion techniques via hands on demonstration; when necessary one-to-one education to physician and nurses in real time<br>12) Project team working with Emergency Department to encourage them to delay catheter placement until person was assessed in the ICU/IMCU<br>13) Daily monitoring including assessment and creation of a plan for catheter removal during multidisciplinary rounds;<br>14) Log maintained by the CNS or Charge RN each patient identifying the indication for the catheter, date inserted and the plan for removal to communicate the information between care givers<br>15) Monitoring the insertion, removal and care components by the unit manager and Clinical Nurse Specialist (CNS). CAUTI and device utilization monitored by Infection Prevention.<br>16) The Critical Care physicians supporting the ICU/IMCU nurses when requesting primary care physician removal of the indwelling urinary catheter if no valid indication for the Foley found.<br>17) Recognition of individual and unit wide positive changes by daily verbal reinforcement and recognition for adoption of new behaviours (informal); and award for clinical staff demonstrating	Incidence of CAUTI	Decrease from pre-intervention CAUTI incidence of 8 to post-intervention rate of 1. Zero CAUTI rate sustained for 394 days.

		early adoption of the culture change, setting expectations for peers and another award for the one unit within the hospital with the greatest decrease in Foley utilization and actual CAUTI events.<br>18) Monthly review of catheter days, CAUTIs, compared against baselines.<br>19) Adherence audited and reinforced with staff who were late adopters.<br>20) The ICU/IMCU celebrating the accomplishment with" Zero" by candy bars and a banner hung in the department recognizing the effectiveness of their hard work		
McCalla et al. (2018);<br>US; a 292-bed community hospital; nurses, respiratory therapists, care managers, physicians, dietary aides, transporters, registrars, physical therapists, housekeeping staff, and technicians	Pre-post test	1) Implementation of automated hand hygiene compliance system (HHCS): a wearable device reminding staff to carry out hand hygiene routine; a yellow and then red light on the device indicating until hand hygiene is performed, at which point the light shines green, assuring patients and anyone at the bedside that HHC was performed.<br>2) Implementation of new catheter insertion kit<br>3)Implementing catheter insertion time-out process<br>4) Introducing procedure for irrigating catheters<br>5) Education of staff on proper catheter insertion and maintenance<br>6) Hospital leadership fostering strong culture of HHC, empowering staff to intervene if non-compliance observed<br>7) Use of user-level compliance data to reinforce efforts<br>8) Use of real time feedback from management	CAUTI rate per 1000 catheter-days;	Decrease from pre-intervention CAUTI rate per 1000 catheter of 2.2 to post-intervention of 1.21 (RRR, 44.8%; 95% CI, 12.7%-65.2%
Miller et al. (2016);<br>US; 2 ICUs; All staff	Pre – post test	1) Teams holding monthly discussions regarding CAUTI incidents<br>2) Teams investigating each infection with help from the infection prevention team and developed interventions to help with barriers<br>3) Nurses provided with education on cleaning patient priori to catheter insertion and every 8-hours<br>4) Team reinforcement of the use of leg straps and early removal<br>5) Team assessment of a written catheter order	CAUTI rate per 1000 catheter-days	Decrease from pre-intervention CAUTI rate per 1000 catheter days of 3 to 1.6 per (year 3); p<0.05); and to 1 (year 4) p<0.001
Mody et al. (2015);<br>US; 12 community-based nursing homes; Nurses' aides, nurses, physicians and infection preventionist	RCT	1) Implementation of pre-emptive barrier precautions including signs placed on the patient room doors, their medical records and nurses station; Barriers precautions also promoted using posters, videos and dance routines<br>2) H.c.p. encouraged to perform hand hygiene before and after providing any care; wearing gowns and gloves when performing care;<br>3) Relevant products placed strategically at intervention sites<br>4) Active surveillance for multidrug resistant organisms and infections, which were reported monthly to staff with the use of graphs, tables and charts.	Incidence of CAUTI; Incidence of CAUTI per catheter days	Intervention group: 56 new episodes of CAUTI over 9413 catheter-days, incidence rate of 5.9 per 1000 catheter-days<br>Control group: 75 new episodes of CAUTI over 8118 catheter-days, incidence rate of 9.2 per 1000 catheter days (95% CI, 0.49-0.99; p=0.45)

		5) Nursing home staff education on key prevention practices and hand hygiene promotion using information pocket card focusing on infection recognition;<br>6) Infection preventionist invited for a half-day conference on surveillance methods for infections;<br>7) Educational programme consisting of 10 modules executed 2-3 months over 3 years including pre-test and post-test questions (targeted at nurses and nurses' aides); education on overview of infection prevention practices, hand hygiene, appropriate indications for device use and device care; sessions presented using DVD, and interactive component including games<br>8) HCW also educated on the indications, technique and duration of effective hand hygiene and appropriate glove and gown use.		
Mody et al. (2017);<br>US; 404 community-based nursing homes; All staff	Pre-post test	1) Implementation of technical bundle highlighting key interventions: catheter removal, aseptic insertion, using regular assessments and feedback, training for catheter care, and incontinence care planning and hydration practices;<br>bundle including infection prevention strategies (e.g. hand hygiene, barrier precautions, and education for infection preventionists, frontline staff, residents, and families), as well as catheter-associated UTI prevention-specific strategies (e.g. prompt removal of unnecessary catheters on admission, adopting evidence-based practices for catheter maintenance and insertion, reducing inappropriate catheter use, and considering alternatives to indwelling urinary catheters<br>2) Technical bundle used as a framework for educational materials , toolkits and interactive strategies<br>3) Importance of the antimicrobial stewardship and appropriate use of diagnostic tests, such as urinalysis and urine culture emphasized<br>4) Education on the technical and socio-adaptive bundles was provided through in-person or virtual learning sessions.<br>6) Monthly coaching calls led by the local organizational lead and national project team faculty providing teams with an opportunity to review data, discuss project implementation, and learn from each other by sharing successes and barriers.<br>7) Onboarding and training webinars at the start of the project preparing facility teams to implement technical and socio-adaptive interventions followed by monthly content webinars presented by faculty experts on the technical and socioadaptive principles of catheter-associated UTI prevention.<br>8) Organizational leads and coaches across the cohorts participating in a separate monthly call to review data trends, learn by sharing,	CAUTI rate per 1000 catheter days; reduction in CAUTI	Decrease from pre-intervention CAUTI rate per 1000 catheter-days of 6.78 to mid-intervention rate of 4.17 and post-intervention rate of 2.63;<br>276 of 368 (75.0%) of the nursing homes reporting at least 40% reduction in CAUTI rates

		identify barriers, and provide feedback to the national project team on needed resources to facilitate implementation.<br>9) Development of an implementation guide, as well as multiple tools, train-the-trainer educational materials to support facility teams at the start and throughout the project		
Mori, C. (2014);<br>US; 150-bed community hospital;<br>Nurses and physicians	Pre-post test	1) Implementation of a nurse driven protocol allowing nurses to remove catheter without a physician order; creation of a decision tree as a quick reference for all staff<br>2) Review of indication or catheter use and alternatives with all nursing staff<br>3) Education provided through on-line learning systems, poster boards and one-to-one sessions<br>4) Education on importance of securing the drainage tubing to the bed sheet; positioning the tubing without dependent loops and maintaining an intact tamper-evident seal.<br>4) Knowledge tests?<br>5) CNS attending nurses and physician staff meetings to address questions related to new protocol.<br>6) Adding insertion criteria to nursing computerised charting and physician order entry systems<br>7) Computerised charting required ongoing assessment by nurses for catheter need and review of indications<br>8) CNS team conducting spontaneous reviews to determine if patient received catheters according to defined criteria- this formed the basis for feedback.	CAUTI rate	Decrease from pre-intervention CAUTI rate of 0.77% to post-(intervention rate of 0.35%
Mullin, K. et al. (2017);<br>US; ICU units in a 1268-bed tertiary care academic centre	Pre-post test	1) Assessment of competency with catheter insertion and maintenance,<br>2) Implementing a closed system for catheters<br>3) Implementation of a nursing driven protocol for catheter removal<br>4) Improved fidelity of electronic documentation of catheters<br>5) Implementation of preservative tubes for specimen collection<br>6) Periodic maintenance audits of catheters.	CAUTI rate per 1000 catheter-days	Decrease from pre-intervention CAUTI rate per 1000 catheter-days of 3 to post-intervention rate of 1.9
Nealon, S. et al (2018);<br>US; a surgical specialty unit in a 802-bed teaching hospital; surgical specialists and nurses	Pre-post test	1) Implementation of the PURCE protocol involving: surgeon deciding in the OR whether catheter is needed and if it is required that it is placed in the OR with a defied time period (less than 48h/more than 48h); in the case of longer duration a yellow band is placed around the drainage tubing to indicate to nurses what to do and a written order is completed; after patient transferred to SSU nurses responsible for identification for the need of catheters and a yellow band; nurses required to remove all catheters without yellow order between 4am and 7 am<br>2) Surgical safety checklist modified to include the news steps and posted in each OR	CAUTI rate	Decrease from pre-intervention rate of 4.29 to post-intervention rate of 1.31

		3) SSU daily audits conducted including possible protocol deviations and patient census; these communicated by the nursing staff to the Infection Prevention Team daily at midnight		
Pashnik, B. et al (2017); US; inpatient, emergency, procedural and surgical areas of a teaching facility; nurses and nursing assistants	Pre-post test	1) Educational intervention emphasising CAUTI prevention as a team effort<br>2) Educational intervention including a) online computer-based learning modules completed independently and b) 2-hour CAUTI prevention competency validation session focusing on utilisation of competency of assessment checklists, facility equipment and hands-on demonstration<br>3) Employment of staff nurses to provide peer education and competency validation to highlight the value of nurses evaluating and influencing clinical practice<br>4) Implementing practice of two nurses inserting catheter<br>5) Learners objectives including : describing CAUTI prevention components; demonstrating catheter insertion and removal techniques; demonstrating proper urine specimen collection techniques; demonstrating correct bladder scanner procedure<br>6) Learners going through 6 competency stations: insertion, care mad maintenance of catheter; specimen collection, daily weights, use of bladder scanners and electronic documentation; and patient education<br>7) CAUTI nurse champions validating peers' knowledge and competency focusing on avoidance of catheters, strict asepsis, catheter care, patient education and interdisciplinary education<br>8) CAUTI champions completing 2-h educational training verifying their competency in CAUTI prevention; these champions then trained their peers, manned the competency stations and validated peers knowledge of the CAUTI bundle	CAUTI rate per 1000 catheter-days	Decrease from pre-intervention CAUTI rate per 1000 catheter-days of 1.3 to post-intervention rate of 0.91 (30% reduction)
Purvis, S. et al (2014); US; academic medical centre; All staff	Pre-post test	1) Modification of existing protocols regarding catheter removal, and indications for straight catheterisation to make them easy to use<br>2) Protocols discussed with lead surgeon and hospitalist to secure their approvals for changes<br>3) Lead surgeon and hospitalist physician discussing protocols with other medical providers to ensure agreement ad to educate on writing orders<br>4) Standardisation of protocols to ensure consistent approach among nurses<br>5) Protocols linked to the clinician order on the electronic health records<br>6) Catheter order formatted to automatically default to the new protocol; de-selection requiring a medical justification.	CAUTI rate per 1000 catheter-days	Decrease from pre-intervention CAUTI rate per 1000 catheter days of 4.2 to post-intervention rate of 3.5 (year 1) and 2.4 (year 2)

		7) All patients with catheters and on bladder management protocol highlighted by a different icon in the electronic health records<br>8) Each unit having CAUTI champion; the initiative endorsed by the Nursing Executive Council<br>9) Education of all CAUTI champions (3h workshop)<br>10) Creation of a CAUTI toolbox on the hospital intranet including educational material<br>11) Daily rounds by the CAUTI champions helping nursing staff to evaluate the necessity for catheter use<br>12) Infection control practitioners providing regular updates on catheter use and CAUTI; rates added to each unit's scorecard.		
Purvis, S. et al. (2016);<br>US; A 592-bed academic tertiary medical centre; nursing and medical staff	Pre-post test	1) Implementation of a hospital leadership walking rounds to discuss CAUTI prevention with front-line staff, analyse next steps, identify successes and areas for improvement and determine whether previous interventions were implemented<br>2) Content of the rounds determined by the Associate Chief Medical Officer and Associate Chief Nursing Office<br>3) Rounds/ interdisciplinary team meetings taking place on a daily basis: various staff invited to the rounds together with leaders<br>4) Rounds involving sharing of data regarding current performance and the 12-month trend<br>5) Identifying staff groups or individuals needing further support<br>6) Support from chair of each department to ensure compliance with protocols	Incidence of CAUTI; CAUTI rate per 100 catheter-days;	Decrease from pre-intervention incidence of CAUTI of 86 to post-intervention incidence of 30; Decrease from pre-intervention CAUTI rate per 1000 catheter-days of 3.1 to post-intervention rate of 1.4 ;
Rhee, Ch. et al (2016);<br>US; academic medical centre; Nurses and physicians	Pre-post test	1) Brainstorming sessions with front line staff in order to choose interventions readily accepted<br>2) Development of a streamlined policy with indications for insertion, continuation and removal of catheter<br>3) Development of a decision tree for staff to use as a decision aid for when to insert a catheter and when to refer to a urologist group<br>4) Standardisation of catheters across hospitals<br>5) Making the process of bulk ordering of nonstandard equipment more difficult<br>6) Creation of exceptions when catheter might be needed<br>7) Education of staff on the new policy and decision tree<br>8) Design of the electronic health records to embed the documentation and the clinical decision support system with indications for insertion, continuation and discontinuation of catheters; this forced staff to assess the indications for catheter on a daily basis<br>9)Elimination of unnecessary fields in the electronic health records and simplification o documentation	Incidence of CAUTI;	Decrease from pre-intervention CAUTI incidence of 135 to 74 (year 1); 59 (year 2) and 25 (year 3)

		10) Allowing nurses to remove catheters when there was no indication for the catheter allowing nurses and physicians to work within guidelines<br>11) Trained observers verifying catheter practices and bundle compliance before and after training videos		
Rhone, C. et al. (2017);<br>US; Emergency Department in a private general hospital; All staff	Pre-post test	1) Insertion of catheter requiring two licensed personnel involving a safety time-out consisting of a pause before inserting the indwelling urinary catheter to confirm catheter appropriateness and review of the proper steps for insertion; catheter inserted by one person while the other responsible for assuring compliance with proper aseptic technique<br>2) Engagement of staff by presentations on the scope of CAUTIs, including the implications to both patients and to the health care system as a whole, from a local (hospital) and a national perspective<br>3) Education of staff by outlining the new process in ED staff education sessions, as well as through handouts, emails, and during shift change huddles<br>4) Introduction of checklist for following proper aseptic technique and reminders of the intent of the project<br>5) Compliance rates monitored and analysis of improvements conducted; staff informed of compliance rates and progress of the project by weekly email updates and periodically in daily huddles.<br>6) Motivating staff to action in order to deliver the highest quality of care to patients.<br>7) Staff accountability encouraged by having the staff sign the checklist used in the new process.<br>8) Timely feedback of data to frontline staff to show whether the goal was being met.<br>9) Feedback from weekly PDSA rapid cycles and constant reinforcement that all insertions must involve 2 personnel.<br>10) Agreement amongst the staff and leadership to keep patients safe and deliver high quality care. 11) Exemplified by explaining the initiative clearly to each patient and their family and allowing for any questions.	Incidence of CAUTI; CAUTI rate per 1000 catheter-days	Decrease from pre-intervention incidence rate of 10 to post-intervention incidence rate of 2 and 3 (year 1 and 2); Decrease from pre-intervention CAUTI rate per 1000 catheter-days of 6.9 to post-intervention rate of 1.7 (p=0.05) ; and 1.6 (p,0.01)
Richards, B. et al. (2017);<br>US; an 18-bed ICU unit in a teaching hospital; Nurses	Pre-post test	1) Creation of "CAUTI Arrest Team" (nurse champions) responsible for implementing the intervention (name to reinforce the seriousness of the problem)<br>2) Nurse champions offering reassurance to staff nurses and addressing their concerns about deficits in care<br>3) Education on importance of daily measurement of body weight to estimate fluid imbalance	Incidence of CAUTI; Standardised Infection Ratio (SIR)	Decrease from pre-intervention incidence rate of 40 and 38 (2-year period) to post-intervention rate of 15.; Decrease from pre-intervention SIR of 2.02 (95% CI, 1.456-2.775) and 2.34 (95% CI, 1.522-3.312) to post-intervention of 1 (Ci 95%, 0.685-1.900); p<0.05, CI 95%, 0.271-0.902/

		4) Nurse champions acknowledging that transition to catheter-free care as difficult and offering support<br>5) Nurses reassured about no individual penalties for occurrences of CAUTI<br>6) Introduction of the programme to staff during "go-live" staff breakfast<br>7) Daily chart audits conducted by charge nurses with help from CAUTI arrest team<br>8)Review of electronic records for all catheterised patients for current physician orders<br>9) Chart audits to check for documentation of perineal and catheter care and daily assessment of catheter<br>10) Implementation of an auto-stop" feature for catheters after 3 days<br>11) Root-case analysis performed for every incidence of CAUTI by CAUTI arrest team with help from infection control nurse<br>12) Education of staff nurses about best practices<br>13) Review of CAUTI incidences during staff meetings<br>14) In-service education about proper insertion techniques including observation of and assistance with insertions<br>15) Education to staff in operation theatre (also including observation and training)<br>16) Purchase of incontinence products and new stool diversion products to replace traditional catheters (with education about it)<br>17) Increased supply to barrier protection products and staff education about it<br>18) Weekly audits identified nurses compliant with interventions- nurses rewarded with gift cards<br>19) Noncompliant nurses provided privately with education but not singled out or penalised.		
Saint et al. (2016);<br>US and Puerto Rico; 926 inpatient units in 603 inpatient units (ICU and non-ICU); All staff	Pre-post test	National Comprehensive Unit-based Safety programme:<br>1)Conducting a daily assessment of the presence and necessity of an indwelling catheters<br>2) Promoting the use of condom catheters, bladder scanners, Intermittent straight catheterization, and accurate measurement of daily weight (all in lieu of indwelling urinary catheters)<br>3) Providing feedback to the unit's nurses and physicians on catheter use and CAUTI rates<br>4) Providing training about any identified gaps in knowledge (tailored training) on urinary management processes (i.e. proper insertion and maintenance using a variety of methods including	Reduction in CAUTI rates;<br>CAUTI rate per 1000 catheter days	CAUTI rate decrease by 22.3%;<br>Decrease from pre-intervention CAUTI rate per 1000 catheter-days of 2.4 to post-intervention rate of 2.05 (incidence rate ratio, 0.86; 95% CI, 0.76 to 0.96; p=0.009

		bedside and electronic (nurses), formal presentations and meetings with one-to-one- discussions for physicians with high use.<br>5) Tools, manuals and checklists provided to help implement the programme.<br>6) Education on the prevention of CAUTIs provided through in-person meetings and webinars (3 over the course of the programme); and coaching calls (on a monthly basis) to provide education, to review data trends, discuss unit specific issues and share best practice.<br>7) Emphasising the importance of aseptic technique and proper maintenance<br>All programmes were customised to each site		
Sampathukamar, P. et al (2016); US; a 213-bed ICU in an academic medical centre; Nurses and physicians	Pre-post test	1)creation of "6 Cs" to make the bundle easy to remember<br>2) Staff encouraged to consider alternatives to catheters each day<br>3) Staff educated on how to select a securement device<br>4) Staff re-educated on the importance of perineal care;<br>5) some units creating bath kits<br>6) Nursing staff reminded about the need to use for catheter system only when medically necessary<br>7) Physicians, nurses and urology staff educated on the use of bladder scanner<br>8) Team creating indications for obtaining urine culture, gaining buy-in from physician leaders and creating educational PowerPoint distributed to all physicians.<br>9) Indications for urine culture ordering added to the electronic health records; indications also reiterated to nurses; cards with indications available on all the units<br>10) Nurses handing out card with urine culture indications to trainees when inappropriate cultures ordered<br>11) Standardisation of documentation of catheter/perineal care<br>12) Implementation of a protocol for ordering bladder scans<br>13) Staff having to select from a list of appropriate indications for urine culture wen placing an order<br>14) CAUTI bundle described and promoted via print, video and online learning<br>15) The campaign's style and colour scheme repeated throughout each educational component to reinforce the education.<br>16) Video with CAUTI champion providing education, able to detect deviation from best practice and offer ways to correct the defect<br>17) Mandatory education of all ICU physicians and all trainees through a PowerPoint presentation; additional in-person CAUTI education at staff meetings	CAUTI rate per 1000 catheter-days	Decrease from pre-intervention CAUTI rate per 1000 catheter days of 2 to post-intervention rate of 0.6 (70% decrease)

		18) A toolkit collating all the resources posted on the Infection Prevention and Control (IPAC) website.		
Scanlon et al. (2017); US; a 814-bed teaching hospital; Nurses, hospital physicians and support staff (Patient care assistants, technicians, physical therapists, and patient transporters)	Pre-post test	1) I Identification and development of CAUTI ambassadors; each ambassador completing education and validation on insertion techniques with demonstration on mannequins; additional instruction on perineal care, maintenance of drainage bags, securing catheter and validating staff on new protocols.<br>2) Education of CAUTI ambassadors and staff on best practice insertion and maintenance guidelines<br>3) Implementation of a nurse-driven straight catheterisation algorithm card<br>4) Interdisciplinary rounds on all patients with catheters in real time to discuss catheter status, opportunities for removal and learning opportunities<br>5) Education of supportive staff including patient care assistants, technicians, physical therapists and patient transporters on best practice catheter maintenance<br>Guidance<br>6) Educational program for hospital physicians, advanced practice providers, nursing leadership and staff to address CAUTI best practice standards<br>7) Monthly analysis and evaluation of every CAUTI case to identify opportunities for improvement; data presented at nursing leadership, quality, and unit-level meetings in a non-threatening environment<br>8) Implementation of CAUTI acronym (C- cause or indication; A- assess for necessity; U- utilise infection prevention measures; T- teach patient and family; I- informatics)<br>9) Purchase of bladder scanners to support implementation of to support nurse to use the algorithm and communicate their findings to provider about patient's plan of care.<br>10) Support from senior leadership to implement emerging best practice from staff<br>11) Consistent access to best practice products assured by supplies service<br>12) Implementation of catheter protocol	Incidence of CAUTI	Decrease in CAUTI incidence by 46% at 12 months and 89% at 18 months
Sutherland, T. et al (2015); US; tertiary medical centre; Nurses and physicians	Pre-post test	1) Measuring catheter use and CAUTI rates for the hospital and later on the individual unit basis; the results recorded and distributed to task force leaders<br>2) Education program for providers<br>3) Implementation of new electronic order set with a decision support<br>4)Provision of daily reminders for catheter removal	CAUTI rate per 1000 catheter-days	Decrease from pre-intervention CAUTI rate per 1000 catheter days of 5.4 to post intervention rate of 1.5 (year 1)

		5) Revision of CAUTI guidelines and instructions		
Ternavasio et al. (2016); Spain; internal medicine department in a university hospital; medical staff, nursing staff and nursing assistants	Pre-post test;	1) Training sessions for the medical staff, nursing staff and nursing assistants on the use of catheters; these sessions included: dissemination of the results obtained during pre-intervention phase; adequate and inadequate indications for the use of UCs; appropriate techniques for placement, maintenance, and timely removal of catheters; and information on the study protocol and the implementation of record-keeping and reminder systems to be implemented during the intervention stage.<br>2) Daily observation of patients with catheters<br>3) Placement of reminder of catheter presence in the patient's progress and treatment record<br>4) Campaign for health care staff with posters and leaflets on ways to optimise the use of catheters and other recommendations<br>5)Feedback of the results through meetings and report	Risk of CAUTI; CAUTI rate per 1000 patients	Decrease in risk of CAUTI (RR: 0.53; 95% CI: 0.30-0.93); p<0.0001; Decrease of CAUTI rate per 1000 patients (RI: 0.52; 95% CI: 0.28-0.94); p= 0.028
Thomas, K. (2016); US; cardiac intensive care and step-down unit in a 536-bed teaching hospital	Pre-post test	1) Skills fair to educate nurses about insertion technique, proper care and indications for catheter use<br>2) Development of nursing round sheet for catheter tracking; nursing rounds taking place three times daily<br>3)Feedback to staff and patients summarising the number of CAUTIs and indwelling catheter days; data also shared at monthly staff meetings	Median number of CAUTIs; CAUTI rate	Decrease from pre-intervention median number of CAUTIs of 2 to post-intervention of 0 (p=0.009); Decrease from pre-intervention CAUTI rate of 10.31 to post intervention of 0 (p=0.005)
Tuttle, J. (2017); US; Two ICU units in a 600-bed tertiary care hospital; All staff	Pre-post test	1)implementation of a nursing driven protocol; nurses empowered to use this tool<br>2)Education to nurses via emails, posters on the unit and discussions about appropriate indications for catheter during bedside conversations using audit tool<br>3) Daily questioning of the need for patient catheter during audit led by unit manager, nurse champion and bedside nurse<br>4) CAUTI prevention lecture delivered by chief nursing officer<br>5) Education of physicians who ordered catheters<br>6) Additional questions in the audit tool enabling tracking catheter orders<br>7) Education and discussions with other departments (ED and surgery) about CAUTI reduction strategies and ensuring standardised use of products<br>8) Monthly CAUTI team meetings to review data, opportunities for improvement and conduct root cause analysis; these findings shared in staff meetings, newsletters and quality bulletin boards<br>9) Celebration of zero CAUTI at the unit<br>10) Tokens given to staff removing catheters which could be exchanged for snacks in the cafeteria<br>11) Provision of alternative to catheter products	Incidence of CAUTI	Decrease from pre-intervention CAUTI incidence from 19 to 10 in year 2-3, and 3 in year 4

		10) Showing evidence to nurses that incontinence not leading to negative patient outcomes<br>12) Clear guidelines on severely ill patients<br>13) Daily conversation with nurses and surgeons at the bedside in order to remove catheter		
Tyson et al. (2018);<br>US; 29-bed Surgical Trauma ICU;<br>Nurses, health care technicians	Pre-post test	1) Unit and nursing ownership of the urinary catheter protocol achieved by implementation of twice-daily CAUTI rounds during which each patient with catheter was evaluated<br>2) Feedback given by peers through the auditing process where opportunities for earlier catheter removal were noted; audit results presented monthly to facility leaders to aid with accountability<br>3) Nurses and physicians education on urine culture stewardship<br>4) Adoption of gray top preservative-containing collection tubes for urine cultures to prevent bacterial overgrowth in the sample	CAUTI rate per 1000 catheter-days; Incidence of CAUTI	Decrease from pre-intervention CAUTI rate per 1000 catheter days of 5.1 to post-intervention rate of 2.0 (p,0.01; RR: 0.38, 95% CI: 0.21-0.65)<br>Decrease from 59 CAUTIs in pre-intervention to 16 in the post-intervention
Umer, A. et al (2016);<br>US; Hospital; Physicians	Pre-post test	1) Implementation of a catheter protocol	CAUTI rate	Decrease from pre-intervention CAUTI rate of 2.6% to post-intervention rate of 1.5% ($p<0.05$)
Quinn, P. (2016);<br>US; hospital;<br>Nurses and physicians	Pre-post test	1) Implementation of "Question the Foley" – a framework to assess the need for a catheter us e by nurses complemented by an algorithm for specific patient circumstances<br>2) Daily assessment of catheter done during day shift<br>3) Chief medical officer approving the plan and being a liaison between nursing and medical staff (for physicians who were not willing to comply with the process)<br>4) Chief of infectious diseases becoming a physician champion- this involved educating physicians about the need to reduce CAUTI; also review of medical<br>5) Design of nurses' documentation, including the criteria for catheter use, in electronic records.<br>5) Nurses having access to customised report showing list of all patients with catheters- used for tracking patients on a daily basis.<br>6) Design of physician documentation and ordering to reflect the nurses' criteria<br>7) reminder message for physicians after 48h (and then every 24h) to review the catheter use<br>8) Targeted education for nurses including large and small group sessions or daily briefing sessions (these included: identifying patients with a catheter on the unit and reviewing key strategies for CAUTI reduction; reports on high incidence of CAUTI across hospital; case presentations including role plays for nurses<br>9) Physicians were provided information about the programme, including reasons for it and what was expected from them, via email and meetings.	CAUTI rate per 1000 catheter-days	Decrease from pre-intervention CAUTI rate per 1000 catheter days of 4.9 to post-intervention rate of 3.9 (year 1), and 0.2 (year 5)

		10) Daily monitoring of documentation- in case of missing documentation emails sent to nursing manager and the nurse counselled<br>11) Report of suspected CAUTIs with tracking when and where the infection was contracted. Each nurse involved in care of that patient sent a letter from the director of nursing informing them that the patient contracted an infection and inviting her to a root analysis meeting. During the meeting each nurse encouraged to identify barriers in or breaks to the prevention initiative.<br>12)Use of silver alloy catheters, implementing the use of securing devices and adherence to proper drainage tube placement<br>13) Educational sessions in large and small group formats including opportunities for staff to identify patients with catheters and review one or two strategies for CAUTI reduction; information about high incidence of CAUTI in the hospital and presentations demonstrating the process of assessing patients, role playing a conversation between a nurse and a physician<br>14) Nurse able to speak to physician if uncertain whether to remove protocol<br>15) Daily assessment of catheter done during day shift to eliminate the phone calls to physicians during off hours		
Youngerman et al. (2018);<br>US; a 2600-bed academic medical centre; nurses and physicians	Pre-post test	1) Training initiative led by the representatives from nurse management and hospital epidemiology to reinforce best practices for proper placement and maintenance of catheters according to guidelines<br>2) Custom module build in electronic records requiring nurses to document the presence and maintenance of catheter every 12h; catheter order or nurse opening an electronic flowsheet to document urinary output triggered the module<br>3) Implementation of real-time electronic tracking of catheters for clinicians (as an optional checklist tool); clinicians able to update the status of each catheter from a drop-down menu with the options: "maintain", "already removed" and "remove today".<br>4) In later phase, the real time electronic tracker becoming a triggered pop-up reminder that needed to be viewed and assessed by a clinician daily; the pop-up was linked to the initiation of a daily progress notes; clinicians not able to complete daily progress note until they have updated the catheter status.<br>5)Catheter order expiring triggering catheter removal by nurses or the need for obtaining a renewal order from physician<br>6) Implementation of a new catheter order requiring clinicians to consider catheter alternatives and to select appropriate criteria for	CAUTI rate per 10 000 patient days;<br>CAUTI rate per 1000 catheter days	Decrease from phase 1 intervention in CAUTI rate per 10 000 patients of 9.06 to post-intervention rate of 1.65 (phase 4); overall decline of 88%;<br>Decrease from phase 1 intervention in CAUTI rate per 1000 catheter days of 4.67 to post-intervention of 1.17 (phase 4) overall decline 74.8%

		catheter placement; clinicians were guided by the branch logic to navigate their decision making process.		
Zubkoff, L. et al (2016); US; 18 Veteran Health Administration facilities nationwide; All staff	Pre-post test	1) Implementation of CAUTI bundle focusing on avoiding catheter use; appropriate catheter use; appropriate insertion technique; appropriate catheter maintenance and timely removal of catheter<br>2) Education of staff via phone, web-based or email on topics including key elements of the bundle, data collection and documentation, lessons learned and best practices , patient engagement and education and barriers to implementing evidence-based intervention<br>3) Teams requiring submission of a senior leader progress report that included overall aims of the project, specific changes implemented during the month and measures of outcome and process being tracked.<br>4) Team cohorts assigned Implementation Coach(quality improvement knowledge) and Clinical Coach (expertise in preventing CAUTI); coaches conducing small-group calls monthly in order to share the completed work and review senior leaders reports, share feedback how to eliminate barriers; feedback from coaches sent to the entire team, including senior leaders.	CAUTI rate per 1000 catheter-days	Decrease from pre-intervention CAUTI rate per 1000 catheter days of 2.37 to post-intervention rate of 1.06 ($p<0.01$)
Zurmehly, J. (2017); US; Long-term acute care hospitals; Nurses and physicians	Pre-post test	1) Implementation of nurse-driven urinary catheter protocol helping nurses to determine the need for the catheter and help with next steps<br>2) Education of nurses about the protocol using emails and posters placed in the break area, monthly meetings<br>3) Nurses completing pre-intervention knowledge questionnaire with feedback received during staff meetings and weekly emails<br>4) Reminders for nurses in the electronic health records every 12 h to determine whether criteria for catheter use still met<br>5) Education of nurses using online self-study module; unit manager responsible for staff education and administrative support of implementation<br>6) System-wide mandatory sessions for all nurses; weekly emails sent until education completed<br>7) Study materials also available in education centre<br>8) Nurses receiving quick reference pocket guide including CAUTI epidemiology, risk factors, definitions, strategies to reduce CAUTI and the new protocol	Incidence of CAUTI; CAUTI rates per 1000 catheter-days	Decrease from pre-intervention CAUTI incidence of 13 to post-intervention of 3 (77%); $p<0.03$;<br>Decrease from pre-intervention CAUTI rate per 1000 catheter-days from 4.82 to post-intervention rate of 1.24 (74%)

Supplementary document 3: The frequency of theoretically congruent BCTs in national and research interventions.

All BCTs paired with key TDF domain as per predefined matrix (5)	BCT frequency in national interventions (5), (Max=11)	% Potential relevant BCTs used at least once	BCT frequency in research interventions, (Max=37)	% Potential relevant BCTs used at least once
**Environmental Context and Resources**				
Restructuring the physical environment<br>Discriminative (learned) cue<br>Prompts/cues<br>Avoidance /changing exposure to the cues for the behaviour<br>Adding objects to the environment<br>Restructuring the social environment	0<br>0<br>1<br>0<br>0<br>1	33%	6<br>0<br>15<br>0<br>22<br>8	66%
**Knowledge**				
Information on health consequences<br>Biofeedback<br>Antecedents<br>Feedback on behaviour<br>Information on social/environmental consequences<br>Information about emotional consequences<br>Salience of consequences	9<br>0<br>0<br>3<br>4<br>1<br>0	57%	7<br>0<br>0<br>27<br>0<br>3<br>2	57%
**Beliefs about Consequences**				
Information about emotional consequences<br>Salience of consequences<br>Covert sensitisation<br>Anticipated regret<br>Information about social/environmental consequences<br>Pros and Cons<br>Vicarious reinforcement<br>Threat<br>Comparative imaging of future outcomes<br>Self-monitoring of behaviour<br>Self-monitoring of outcome of behaviour<br>Information on health consequences<br>Feedback on behaviour<br>Biofeedback<br>Feedback on outcome of behaviour<br>Persuasive communication (credible source)	1<br>0<br>0<br>0<br>4<br>0<br>0<br>0<br>0<br>5<br>3<br>9<br>3<br>0<br>3<br>3	50%	3<br>2<br>0<br>0<br>0<br>0<br>0<br>0<br>0<br>8<br>0<br>7<br>27<br>0<br>13<br>7	43%
**Social Influences**				
Social comparison<br>Social support unspecified	1<br>0	50%	0<br>3	70%

Social support emotional	0		1	
Social support practical	4		13	
Information about others approval	0		5	
Vicarious consequences/reinforcement	0		0	
Restructuring the social environment	1		8	
Identification of self as a model	1		0	
Social reward	0		4	
Demonstration of behaviour	3		17	
Memory, Attention and Decision Making				
Self-monitoring of behaviour	5	100%	8	75%
Self-monitoring of outcome of behaviour	3		0	
Action planning	1		9	
Prompt and cues	1		15	
Social Professional Role and Identity				
Social support unspecified	0	33%	3	100%
Social support emotional	0		1	
Social support practical	4		13	

Supplementary Document 4: List of intervention components included in the survey.

Theme/Barrier (Based on CAUTI Report)	TDF Domains	Intervention	BCTs delivered	Sources of the suggested intervention
Limited and inconsistent documentation and records; Transitions of care; Lack of information regarding placement and duration of catheter insertion	Environmental Context and Resources; Knowledge	Standardised nationwide computer-based documentation, accessible across healthcare sectors, requiring person initiating urinary catheterisation to insert details such as date of catheter insertion, reason for catheterisation, an action plan for review and removal and details of difficult catheterisation (if relevant). Provided when transferring patients across settings	Restructuring the physical environment, Prompts/cues, Action planning, Restructuring the social environment.	Previous CAUTI report, rapid review and stakeholders
CAUTI guidelines not perceived as relevant across hospital departments/ settings	Social Professional Role and Identity	Ensure availability of setting and profession specific guidelines which are in agreement and which include examples of how to adapt to local contexts where possible.	Adding objects to the environment/ Restructuring the physical environment, Social support practical	Previous CAUTI report, stakeholders, and the project team
Poor urinary catheter insertion technique	Skills	Provision of face-to-face training for nurses in catheter insertion, maintenance and removal	Instruction on how to perform a behaviour; Behavioural demonstration	Stakeholders
Unavailability of medical alternatives to urinary catheterisation; Lack of knowledge of how to manage patients without catheters	Environmental Context and Resources; Knowledge	Increase availability and visibility of alternatives to catheters including; continence pads, commodes, continence sheaths and female urinary devices (e.g. funnel) to encourage wider use.	Restructuring the physical environment; Behavioural substitution; Instruction on how to perform a behaviour	Rapid review, stakeholders, project team and the steering group
Unavailability of medical alternatives to urinary catheterisation; Lack of knowledge of how to manage patients without catheters	Environmental Context and Resources; Knowledge	Promotion/ wider implementation of intermittent urinary catheterisation as an alternative to indwelling urinary catheters complemented by staff training on clinical indications and appropriate use of intermittent catheterisation.	Adding objects to the environment, Behavioural substitution, Instruction on how to perform a behaviour, Behavioural practice/rehearsal	Stakeholders and steering group
Unavailability of medical alternatives; Lack of knowledge of how to manage patients without catheters	Environmental Context and Resources; Knowledge	Provision of bladder scanners, accompanied by staff training on how to use scanners, to aid decisions in relation to problems with urinary retention.	Adding objects to the environment, Behavioural substitution, Instruction on how to perform a behaviour, Behavioural practice/rehearsal	Rapid review, stakeholders and the steering group
Transitions of care; Pre-emptively deciding to insert catheters due to likely subsequent catheterisation	Environmental Context and Resources; Memory, Attention and Decision Making	Creating the rule that staff transferring catheterised patients to another setting, check/review the need for a catheter with the receiving team. This rule could be prompted by a checklist for discharge/admission of patients to another setting.	Restructuring the social environment, Prompts and Cues, Action planning	Previous CAUTI report

Physicians dictate nurses' practice; Lack of peer support and buy-in	Social Influences	Introduction of "CAUTI Champions" (nurses and doctors). Champions role model how to manage patient/carer requests for catheter, lead on staff education and provide practical support for colleagues wanting to support patients to TWOC (trial without catheter).	Restructuring the social environment; Social support practical;	Rapid review, stakeholders, project team and the steering group
The issue of catheterising for convenience; Perceived severity of CAUTI; Lack of awareness of the risks associated with use of urinary catheter	Beliefs about Consequences; Knowledge	Intervention to persuade staff of benefits of not using catheters for both patients (e.g. loss of mobility, bed sores, lower risk of infection) and staff (e.g. fewer patients developing infection, improved patient outcomes, lower costs). Reassure staff that not using catheters does not lead to suboptimal care and reframing severity of CAUTI as patient safety issue with a story of a patient who contracted CAUTI.	Information about health consequences, Salience of consequences, Instruction on how to perform a behaviour, Social support unspecified, Anticipated regret	Rapid review, stakeholders, project team and the steering group
Requests from patients and their carers	Social Influences	Before inserting catheters, staff are required to inform patients and relatives about pros and cons of catheters, risks associated with catheter use, including sepsis and antibiotic resistance as well as the importance of hydration (with or without written resources) and record that this has been explained to patients.	Information about health consequences; Salience of consequences; Pros and cons.	Previous CAUTI report and stakeholders
Perceived severity of CAUTI; Lack of awareness of risks associated with catheter use	Beliefs about Consequences; Knowledge	Provide feedback to staff on any patients with CAUTI in their care. Provide case review and analysis of care pathway to identify and feedback where staff could have protected the patient and stopped or reviewed catheter use.	Feedback on behaviour, Feedback on outcome of behaviour; Problem solving	Rapid review, stakeholders, project team and the steering group
Challenging practice norms for catheterisation in specific patient groups; Absence of standard CAUTI diagnostics criteria to help decision making	Social Influences; Memory, Attention and Decision Making	Compare and feedback rates of catheterisation across similar settings with corresponding rates of infection, if suitable metric is developed.	Social comparison	Previous CAUTI report, steering group and the project team
Perceived severity of CAUTI; Lack of awareness of risks associated with catheter use	Beliefs about Consequences; Knowledge	Cascade a Patient Safety Alert for CAUTI.	Adding objects to the environment; Feedback on outcome of behaviour	Solution suggested by the project team
Lack of information regarding placement and duration of catheter	Knowledge	Implementation of a reminder system to prompt staff to review catheter use, for example a sticker on patient notes or a computerised prompt.	Adding objects to the environment, Prompt and cues	Rapid review, stakeholders, project team and the steering group
Lack of information regarding placement and duration of catheter.	Knowledge	Daily review of number of catheters on ward including rates of catheters fitted, removed and in situ; numbers displayed on ward information board.	Feedback on behaviour	Rapid review, project team and the steering group

Transitions of care	Environmental Context and Resources	Rule that patients coming from A & E/ICU/surgery have immediate catheter review (with catheter removal where possible) when they arrive on the ward and then review every 24 hours.	Restructuring the social environment; Action planning	Previous CAUTI report, project team and the steering group
Limited and inconsistent documentation and records; Transitions of care	Environmental Context and Resources	Shift manager allocates a staff member (doctor or nurse) to be responsible for review of urinary catheterisation decisions and to be responsible for overseeing/ensuring documentation of catheter decisions/ward transfers.	Restructuring the social environment	Project team and the steering group
Lack of peer support and buy-in	Social Influences	Trust-wide multi-disciplinary commitment to catheter guidelines. Leaders and senior members of staff denouncing catheterising for convenience.	Information about others approval; Restructuring the social environment	Previous CAUTI report, project team and the steering group
Lack of information regarding placement and duration of catheter	Knowledge	GP is given information that a patient is catheterised and the catheter review date when patient is discharged from another care setting and this is clear on patient records. GP is prompted to check plan for catheter removal when seeing a patient and refer on to appropriate person.	Restructuring the social environment; Adding objects to the environment; Prompts and Cues	Stakeholders, project team and the steering group
Unavailability of medical alternatives to urinary catheterisation; Lack of knowledge of how to manage patients without catheters	Environmental Context and Resources; Knowledge	District nurses to be provided with guidance and support to TWOC - 'trial without catheter'.	Behavioural substitution, Social support practical, Instruction on how to perform a behaviour	Stakeholders, project team and the steering group

Supplementary Document 5: Relevance and APEASE scores for all intervention components in primary/community care.

**Intervention component**	**Deemed relevant by n: (max no of participants =11)**	**A**	**P**	**E**	**A**	**S**	**E**	**Total score (max= 66)**	**APEASE %**
Ensure availability of setting and profession specific guidelines which are in agreement and which include examples of how to adapt to local contexts where possible.	11/11	6/11	5/11	6/11	9/11	9/11	7/11	42/66	63%
Creating the rule that staff transferring catheterised patients to another setting, check/review the need for a catheter with the receiving team. This rule could be prompted by a checklist for discharge/admission of patients to another setting.	9/11	8/11	8/11	8/11	8/11	9/11	7/11	48/66	72%
Before inserting catheters, staff are required to inform patients and relatives about pros and cons of catheters, risks associated with catheter use, including sepsis and antibiotic resistance as well as the importance of hydration (with or without written resources) and record that this has been explained to patients.	9/11	8/11	8/11	7/11	8/11	8/11	6/11	45/66	68%
Intervention to persuade staff of benefits of not using catheters for both patients (e.g. loss of mobility, bed sores, lower risk of infection) and staff (e.g. fewer patients developing infection, improved patient outcomes, lower costs). Reassure staff that not using catheters does not lead to suboptimal care and reframing severity of CAUTI as patient safety issue with a story of a patient who contracted CAUTI.	9/11	6/11	5/11	7/11	7/11	8/11	5/11	38/66	57%
Standardised nationwide computer-based documentation, accessible across healthcare sectors, requiring person initiating urinary catheterisation to insert details such as date of catheter insertion, reason for catheterisation, an action plan for review and removal and details of difficult catheterisation (if relevant). Provided when transferring patients across settings.	8/11	3/11	3/11	8/11	8/11	8/11	7/11	37/11	56%
Introduction of "CAUTI Champions" (nurses and doctors). Champions role model how to manage patient/carer requests for catheter, lead on staff education and provide practical support for colleagues wanting to support patients to TWOC (trial without catheter).	8/11	5/11	3/11	7/11	6/11	8/11	4/11	33/66	50%
Provision of face-to-face training for nurses in catheter insertion, maintenance and removal.	8/11	5/11	3/11	7/11	7/11	6/11	4/11	32/66	48%
Promotion/ wider implementation of intermittent urinary catheterisation as an alternative to indwelling urinary catheters complemented by staff training on clinical indications and appropriate use of intermittent catheterisation.	8/11	4/11	2/11	6/11	4/11	6/11	3/11	25/66	37%
Provide feedback to staff on any patients with CAUTI in their care. Provide case review and analysis of care pathway to identify and feedback where staff could have protected the patient and stopped or reviewed catheter use.	8/11	3/11	1/11	5/11	3/11	7/11	4/11	23/66	34%

Implementation of a reminder system to prompt staff to review catheter use, for example a sticker on patient notes or a computerised prompt.	7/11	6/11	5/11	7/11	8/11	7/11	4/11	37/66	56%
Increase availability and visibility of alternatives to catheters including; continence pads, commodes, continence sheaths and female urinary devices (e.g. funnel) to encourage wider use.	7/11	5/11	4/11	5/11	5/11	7/11	4/11	30/66	45%
Compare and feedback rates of catheterisation across similar settings with corresponding rates of infection, if suitable metric is developed.	7/11	5/11	3/11	6/11	5/11	5/11	4/11	28/66	42%
GP is given information that a patient is catheterised and the catheter review date when patient is discharged from another care setting and this is clear on patient records. GP is prompted to check plan for catheter removal when seeing a patient and refer on to appropriate person.	6/11	7/11	5/11	6/11	6/11	7/11	4/11	35/66	53%
District nurses to be provided with guidance and support to TWOC - 'trial without catheter'.	6/11	4/11	4/11	6/11	6/11	5/11	5/11	30/66	45%
Provision of bladder scanners, accompanied by staff training on how to use scanners, to aid decisions in relation to problems with urinary retention.	6/11	3/11	3/11	6/11	5/11	6/11	4/11	27/66	40%
Cascade a Patient Safety Alert for CAUTI.	6/11	3/11	3/11	6/11	4/11	3/11	3/11	22/66	33%

Supplementary Document 6: Relevance and APEASE scores for all intervention components in secondary care.

Intervention component	Deemed relevant by n: (max=9)	A	P	E	A	S	E	Total score (max =54)	APEASE %
Ensure availability of setting and profession specific guidelines which are in agreement and which include examples of how to adapt to local contexts where possible.	9/9	4/9	4/9	5/9	7/9	7/9	6/9	33/54	61%
Standardised nationwide computer-based documentation, accessible across healthcare sectors, requiring person initiating urinary catheterisation to insert details such as date of catheter insertion, reason for catheterisation, an action plan for review and removal and details of difficult catheterisation (if relevant). Provided when transferring patients across settings.	8/9	3/9	/9	8/9	8/9	8/9	7/9	37/54	68%
Provision of bladder scanners, accompanied by staff training on how to use scanners, to aid decisions in relation to problems with urinary retention.	7/9	5/9	6/9	7/9	7/9	7/9	5/9	37/54	68%
Before inserting catheters, staff are required to inform patients and relatives about pros and cons of catheters, risks associated with catheter use, including sepsis and antibiotic resistance as well as the importance of hydration (with or without written resources) and record that this has been explained to patients.	7/9	6/9	6/9	6/9	7/9	6/9	6/9	37/54	68%
Creating the rule that staff transferring catheterised patients to another setting, check/review the need for a catheter with the receiving team. This rule could be prompted by a checklist for discharge/admission of patients to another setting.	7/9	6/9	6/9	6/9	6/9	7/9	5/9	36/54	66%
Introduction of "CAUTI Champions" (nurses and doctors). Champions role model how to manage patient/carer requests for catheter, lead on staff education and provide practical support for colleagues wanting to support patients to TWOC (trial without catheter).	7/9	5/9	3/9	7/9	6/9	7/9	5/9	33/54	61%
Intervention to persuade staff of benefits of not using catheters for both patients (e.g. loss of mobility, bed sores, lower risk of infection) and staff (e.g. fewer patients developing infection, improved patient outcomes, lower costs). Reassure staff that not using catheters does not lead to suboptimal care and reframing severity of CAUTI as patient safety issue with a story of a patient who contracted CAUTI.	7/9	5/9	4/9	6/9	5/9	6/9	5/9	31/54	57%
Increase availability and visibility of alternatives to catheters including; continence pads, commodes, continence sheaths and female urinary devices (e.g. funnel) to encourage wider use.	7/9	5/9	4/9	5/9	5/9	7/9	4/9	30/54	55%
Provision of face-to-face training for nurses in catheter insertion, maintenance and removal.	6/9	4/9	5/9	6/9	6/9	6/9	5/9	32/54	59%
Trust-wide multi-disciplinary commitment to catheter guidelines. Leaders and senior members of staff denouncing catheterising for convenience.	6/9	6/9	5/9	5/9	5/9	6/9	5/9	32/54	59%
Daily review of number of catheters on ward including rates of catheters fitted, removed and in situ; numbers displayed on ward information board.	6/9	5/9	5/9	5/9	5/9	6/9	5/9	31/54	57%
Implementation of a reminder system to prompt staff to review catheter use, for example a sticker on patient notes or a computerised prompt.	6/9	4/9	4/9	6/9	6/9	5/9	4/9	29/54	53%

Shift manager allocates a staff member (doctor or nurse) to be responsible for review of urinary catheterisation decisions and to be responsible for overseeing/ensuring documentation of catheter decisions/ward transfers.	6/9	5/9	5/9	4/9	4/9	6/9	5/9	29/54	53%
Rule that patients coming from A & E/ICU/surgery have immediate catheter review (with catheter removal where possible) when they arrive on the ward and then review every 24 hours.	6/9	4/9	4/9	5/9	5/9	6/9	5/9	29/54	53%
Provide feedback to staff on any patients with CAUTI in their care. Provide case review and analysis of care pathway to identify and feedback where staff could have protected the patient and stopped or reviewed catheter use.	6/9	2/9	2/9	4/9	4/9	6/9	4/9	22/54	40%
Cascade a Patient Safety Alert for CAUTI.	5/9	4/9	4/9	5/9	5/9	4/9	4/9	26/54	48%
Compare and feedback rates of catheterisation across similar settings with corresponding rates of infection, if suitable metric is developed.	5/9	4/9	2/9	4/9	4/9	4/9	4/9	22/54	40%
Promotion/ wider implementation of intermittent urinary catheterisation as an alternative to indwelling urinary catheters complemented by staff training on clinical indications and appropriate use of intermittent catheterisation.	5/9	3/9	1/9	5/9	3/9	4/9	3/9	19/54	35%

Supplementary Document 7: Relevance and APEASE scores for all intervention components in care homes.

**Intervention component**	**Deemed relevant by n: (max=10)**	**A**	**P**	**E**	**A**	**S**	**E**	**Total score (max 60)**	**% APEASE**
Creating the rule that staff transferring catheterised patients to another setting, check/review the need for a catheter with the receiving team. This rule could be prompted by a checklist for discharge/admission of patients to another setting.	9/10	8/10	7/10	8/10	8/10	9/10	7/10	47/60	78%
Before inserting catheters, staff are required to inform patients and relatives about pros and cons of catheters, risks associated with catheter use, including sepsis and antibiotic resistance as well as the importance of hydration (with or without written resources) and record that this has been explained to patients.	9/10	8/10	7/10	8/10	9/10	8/10	7/10	47/60	78%
Intervention to persuade staff of benefits of not using catheters for both patients (e.g. loss of mobility, bed sores, lower risk of infection) and staff (e.g. fewer patients developing infection, improved patient outcomes, lower costs). Reassure staff that not using catheters does not lead to suboptimal care and reframing severity of CAUTI as patient safety issue with a story of a patient who contracted CAUTI.	9/10	5/10	3/10	8/10	7/10	8/10	6/10	37/60	61%
Ensure availability of setting and profession specific guidelines which are in agreement and which include examples of how to adapt to local contexts where possible	9/10	4/10	2/10	5/10	7/10	7/10	6/10	31/60	51%
Standardised nationwide computer-based documentation, accessible across healthcare sectors, requiring person initiating urinary catheterisation to insert details such as date of catheter insertion, reason for catheterisation, an action plan for review and removal and details of difficult catheterisation (if relevant). Provided when transferring patients across settings.	8/10	3/10	3/10	8/10	8/10	8/10	7/10	37/60	61%
Increase availability and visibility of alternatives to catheters including; continence pads, commodes, continence sheaths and female urinary devices (e.g. funnel) to encourage wider use.	8/10	6/10	4/10	3/10	6/10	6/10	8/10	33/60	55%
Introduction of "CAUTI Champions" (nurses and doctors). Champions role model how to manage patient/carer requests for catheter, lead on staff education and provide practical support for colleagues wanting to support patients to TWOC (trial without catheter).	8/10	3/10	2/10	7/10	6/10	7/10	5/10	30/60	50%
Provide feedback to staff on any patients with CAUTI in their care. Provide case review and analysis of care pathway to identify and feedback where staff could have protected the patient and stopped or reviewed catheter use.	8/10	2/10	1/10	6/10	4/10	7/10	5/10	25/60	41%
Implementation of a reminder system to prompt staff to review catheter use, for example a sticker on patient notes or a computerised prompt.	6/10	5/10	4/10	5/10	5/10	6/10	4/10	29/60	48%

Provision of face-to-face training for nurses in catheter insertion, maintenance and removal.	6/10	3/10	3/10	5/10	6/10	5/10	4/10	26/60	43%
Cascade a Patient Safety Alert for CAUTI.	6/10	4/10	4/10	5/10	4/10	4/10	4/10	25/60	41%
Compare and feedback rates of catheterisation across similar settings with corresponding rates of infection, if suitable metric is developed.	6/10	3/10	1/10	4/10	4/10	4/10	4/10	20/60	33%
Promotion/ wider implementation of intermittent urinary catheterisation as an alternative to indwelling urinary catheters complemented by staff training on clinical indications and appropriate use of intermittent catheterisation.	5/10	2/10	0/10	5/10	4/10	4/10	3/10	18/60	30%
Provision of bladder scanners, accompanied by staff training on how to use scanners, to aid decisions in relation to problems with urinary retention.	4/10	1/10	0/10	4/10	3/10	4/10	3/10	15/60	25%

Supplementary Document 8: Search terms- rapid review.

(Example from Medline Search Terms (1 January 2014-23 October 2018)

1 Catheter-Related Infections/
2 ((catheter related or catheter associated) adj2 infection?).ti,ab.
3 catheter*.ti.
4 catheters/
5 1 or 2 or 3 or 4
6 exp Urinary Tract Infections/
7 (((urin* or bladder*) adj2 infection?) or cauti or cautis or uti or utis or bacteriuria or pyuria or cystitis).ti,ab.
8 6 or 7
9 5 and 8
10 catheters, indwelling/ or urinary catheters/
11 ((urin* or bladder? or indwelling) adj3 catheter*).ti,ab.
12 10 or 11
13 infection*.mp.
14 12 and 13
15 9 or 14
16 Catheter-Related Infections/pc or exp Urinary Tract Infections/pc
17 Infection Control/
18 ((infection? or cauti or cautis or uti or utis or bacteriuria or pyuria or cystitis) adj5 (reduc* or decreas* or prevent*)).ti,ab.
19 ((infection? or cauti or cautis or uti or utis or bacteriuria or pyuria or cystitis) adj2 rate?).ti,ab.
20 (infection? adj2 control*).ti,ab.
21 prevent*.ti.
22 16 or 17 or 18 or 19 or 20 or 21
23 (educat* or train* or learn* or teach* or class* or workshop* or lecture? or module? or self-study or studying).ti,ab.
24 (program* or project? or intervention or initiative?).ti,ab.
25 (competenc* or knowledge* or skill?).ti,ab.
26 (behav* adj3 (chang* or modif* or improv*)).ti,ab.
27 (practice adj3 (chang* or modif* or improv*)).ti,ab.
28 (quality adj3 (improv* or manag* or chang*)).ti,ab.
29 exp Health Personnel/ed [Education]
30 exp education, continuing/ or exp inservice training/
31 exp Professional Competence/
32 practice patterns, nurses'/ or practice patterns, physicians'/
33 23 or 24 or 25 or 26 or 27 or 28 or 29 or 30 or 31 or 32
34 15 and 22 and 33
35 limit 34 to "reviews (maximizes specificity)"
36 limit 35 to (english language and yr="2000 -Current")
37 34 not 36
38 limit 37 to (english language and yr="2000 -Current")

Supplementary Document 9: List of most frequently identified barriers[5].

TDF domain	Barriers
**Knowledge**	Lack of knowledge of clinical guidelines and local procedural documents
	Lack of information regarding placement and duration of catheter insertion
	Lack of awareness of the risks associated with use of urinary catheters
	Lack of knowledge of how to manage patients without catheterisation
	Knowledge of how to manage bacterial infections resulting from urinary catheterisation
**Memory, Attention and Decision Making**	Pre-emptively deciding to insert a catheter due to likely subsequent catheterisation
	Catheterisation decisions based on non-medical criteria
	Patient symptoms prompt investigation and treatment of possible CAUTI
	Absence of standardised CAUTI diagnostic criteria to help decision-making
	Reminders and prompts
**Behavioural regulation**	Audit and feedback on CAUTI metrics
	Inconsistent monitoring of compliance with guidelines
**Skills**	Poor urinary catheter insertion technique
**Environmental Context and Resources**	Limited and inconsistent documentation and records
	Transitions of care
	Lack of time to perform alternatives to urinary catheterisation
	Unavailability of medical alternatives to urinary catheterisation
	Choice and availability of urinary catheters
**Social Influences**	Requests from patients and their carers
	Lack of peer support and buy-in
	Physicians dictate nurses' practice
	Cultural norms regarding standard catheterisation practice for specific patient groups
**Social Professional Role and Identity**	Local champions
	Having a Hospital Epidemiologist in post
	Acceptance of responsibility for urinary catheterisation decision making
	CAUTI guidelines not perceived as relevant across Hospital departments
	Nurses leading change in urinary catheterisation practice
**Beliefs about Consequences**	Convenience and ease of monitoring
	Perceived severity of CAUTI
	Lack of perceived benefits to interventions targeting CAUTI
	Improved patient hygiene
	Pros and cons of reusable catheters
**Beliefs about Capabilities**	Nurse empowerment
	Confidence in investigating and managing CAUTI

Goals	CAUTI is not a priority
